# Role of SNAREs in Unconventional Secretion—Focus on the VAMP7-Dependent Secretion

**DOI:** 10.3389/fcell.2022.884020

**Published:** 2022-06-16

**Authors:** Somya Vats, Thierry Galli

**Affiliations:** ^1^ Institute of Psychiatry and Neuroscience of Paris (IPNP), INSERM U1266, Membrane Traffic in Healthy and Diseased Brain, Université Paris Cité, Paris, France; ^2^ GHU PARIS Psychiatrie & Neurosciences, Paris, France

**Keywords:** unconventional protein secretion, VAMP7, SNARE, cancer, neurodegeneration

## Abstract

Intracellular membrane protein trafficking is crucial for both normal cellular physiology and cell-cell communication. The conventional secretory route follows transport from the Endoplasmic reticulum (ER) to the plasma membrane via the Golgi apparatus. Alternative modes of secretion which can bypass the need for passage through the Golgi apparatus have been collectively termed as Unconventional protein secretion (UPS). UPS can comprise of cargo without a signal peptide or proteins which escape the Golgi in spite of entering the ER. UPS has been classified further depending on the mode of transport. Type I and Type II unconventional secretion are non-vesicular and non-SNARE protein dependent whereas Type III and Type IV dependent on vesicles and on SNARE proteins. In this review, we focus on the Type III UPS which involves the import of cytoplasmic proteins in membrane carriers of autophagosomal/endosomal origin and release in the extracellular space following SNARE-dependent intracellular membrane fusion. We discuss the role of vesicular SNAREs with a strong focus on VAMP7, a vesicular SNARE involved in exosome, lysosome and autophagy mediated secretion. We further extend our discussion to the role of unconventional secretion in health and disease with emphasis on cancer and neurodegeneration.

## The Secretory Pathway

The secretory pathway deals with synthesis and delivery of proteins either membrane associated or not into the extracellular space and as receptors at the cell surface (Popescu, 2012). Secreted proteins which make up the secretome account for 9–15% of the total human proteome and serve major roles in cellular physiology, pathology and intercellular communication. Depending on the mode of secretion, the secretory pathway can be either conventional or unconventional. In this review we will focus on the molecular and cellular mechanisms of secretion in the extracellular space, and refer to other reviews regarding the transport of receptors to the cell surface.

## Conventional Protein Secretion

Classical or conventional secretory pathway begins at the Endoplasmic reticulum (ER) where new secreted protein synthesis occurs. A major early event in this route is the insertion of proteins destined to secretion, such as proteins of the extracellular matrix, cytokines, peptidic hormones and neuropeptides, into the lumen of the ER. This translocation is mediated by a short hydrophobic sequence at the amino-terminus called leader sequence or signal peptide (Viotti, 2016). Hormone and neurotransmitter receptors, adhesion molecules and ionic pumps of the plasma membrane are additionally equipped with one or several transmembrane domains in addition of the leader sequence. These newly synthesized proteins then move to the Golgi apparatus (GA), after passing through and ER-Golgi intermediate compartment (ERGIC). After the GA, proteins destined to secretion are packed in secretory vesicles which subsequently transport them towards the plasma membrane. Finally, these secretory vesicles fuse with the plasma membrane thereby releasing their contents in the extracellular space. Conventional secretion can be constitutive or regulated, referring in general to a regulation by intracellular calcium concentration, sometimes to other second messengers (Benham, 2012). Constitutive secretion such as release of collagen, proteoglycans and interleukins occurs in all cells constantly while regulated secretion such as release of peptidic hormones like insulin or neuropeptides occurs in some specialized animal cells upon signaling cue. In the ER, secreted proteins undergo several important modifications: cleavage of the leader sequence, proteolysis and glycosylations. From the ER, the proteins exit in COP-II vesicles and take the route to the GA where they undergo further additional reactions of glycosylation and deglycosylation, sulfatation or phosphorylation (Ungar, 2009; Huang and Wang, 2017). These modifications occur in a well-ordered sequential manner from the cis-to the medial-to the trans-Golgi network (TGN). Secreted proteins are packaged into secretory vesicles at the exit of the TGN, in mechanisms involving different types of adaptors (Di Martino et al., 2019; Tan and Gleeson, 2019). The resulting secretory granules can further mature with a condensation of their content and the retrieval of some of its components (Hammel et al., 2010). Secretory vesicles are then transported towards the plasma membrane where they finally fuse (Burgess and Kelly, 1987; Benham, 2012; Viotti, 2016). Thul *et al.*, in 2017 published a subcellular map of the human proteome in which they identified 2,918 proteins secreted by the conventional secretion by using bioinformatic tools to score for signal peptide and transmembrane domains (Thul et al., 2017). Uhlen *et al.*, further enriched this knowledge in their comprehensive report on human secretome in which they tried to decipher the destinations of actively secreted human proteins (Uhlén et al., 2019).

## Unconventional Protein Secretion

Work published over the past decade unearthed alternative routes which can bypass the need for passage through the GA and has been collectively termed as Unconventional protein secretion (UPS). Most of the proteins secreted by UPS are leaderless proteins, i.e., they lack targeting signal sequences and their mode of secretion has been classified into four classes: Type I, Type II and Type III and Type IV secretion.

**FIGURE 1:**
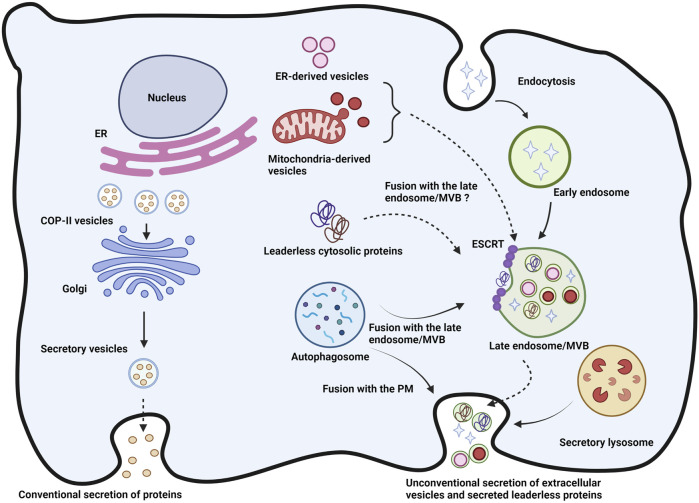
Figure of unconventional vs. conventional secretions: Newly synthesized proteins from the ER and exported in COP-II vesicles towards the Golgi apparatus wherein they are packed in secretory vesicles for export *via* the conventional secretory pathway. Unconventional secretion, in particular the Type III secretion represented here involves packaging of cytosolic proteins as well as the possible targeting of autophagic, ER-derived and mitochondria-derived vesicles to the multivesicular bodies (MVBs) or late endosomes. The resultant intraluminal vesicle can then fuse with the plasma membrane and release exosomes and other mixed content. Autophagosomes and secretory lysosomes can fuse with the plasma membrane directly to release their content in the extracellular space. MVB: Multivesicular body; ILV: Intraluminal vesicle; PM: Plasma membrane. Role of SNAREs and their partners in unconventional secretion.

Type I secretion is lipidic pore-mediated translocation of cytoplasmic proteins across the plasma membrane; Type II is ABC transporter-based secretion of acylated proteins, and Type III is packaging of cytoplasmic proteins in vesicles of autophagosomal/endosomal origin which fuse with the plasma membrane and release these proteins in the extracellular space. Type IV UPS involves transmembrane proteins with or without signal sequences which pass the ER and reach the plasma membrane for secretion without going through the Golgi apparatus. Type I and Type II secretion are non-vesicular and non-SNARE protein dependent whereas Type III and Type IV dependent on vesicles and on SNARE proteins (Rabouille, 2017; Dimou and Nickel, 2018). Figure 1 summarizes the general features of conventional and unconventional secretion. Most of the UPS pathways seem to be triggered by stress conditions such as nutrient deprivation, ER stress, mechanical stress or inflammation and also in the context of cell growth (Wojnacki et al., 2020). This precludes the proper functioning of the ER-Golgi secretion system thereby aggravating the need of an alternate secretory system. The need to bypass the ER-Golgi pathway also arises for proteins such as Fibroblast growth factor 2 (FGF2) which could be rendered biologically inactive upon undergoing glycosylation (Rabouille, 2017; Dimou and Nickel, 2018). Finally, unconventional protein secretion is a way for secretion of proteins such as High mobility group box1 (HMGB1) and acyl-CoA binding protein (ACBP) which have different intracellular and extracellular functions in physiological versus stress conditions (Gardella et al., 2002; Duran et al., 2010). The secretion of extracellular vesicles is one of the most prominent unconventional secretory mechanisms unveiled in the last decades. It was initially discovered in cancer cells as the exfoliation of membrane ecto-enzymes in the form of micro-vesicles referred to as exosomes (Trams et al., 1981). Since then, the formation and release of extracellular vesicles has redefined many rules of secretory mechanism. Indeed, exosomes are formed by invagination of the limiting membrane of late endosomes, defining intraluminal vesicles, in a mechanism which require the ESCRT machinery (Adell et al., 2014). Exosomes are small and rather homogenous whereas other types of larger extracellular vesicles might be more heterogenous in nature as it was recently shown in the case of the release of amphisomes, a mixed secretory organelle with both autophagosomal and late endosomal origin (Jeppesen et al., 2019). The situation is further complicated by the occurrence of microvesicles or ectosomes which originate from the plasma membrane and share biophysical properties with exosomes but are still distinct extracellular vesicles (Mathieu et al., 2021). The recent work from our laboratory and the Demetriades and Debnath laboratories are coherent with the notion that late endosomes and autophagosomes are connected and that secretion might involve a mixed content (Leidal et al., 2020; Wojnacki et al., 2020; Nüchel et al., 2021). During their formation, intraluminal vesicles capture components of the limiting membrane of late endosomes such as tetraspanins (CD81, CD63) and cytosolic proteins. Cytosolic proteins captured into intraluminal vesicles lack a leader sequence like that of secreted peptides in the conventional route and there is not a defined consensus sequence that would target cytosolic proteins to intraluminal vesicles. Thus, whether intraluminal vesicles capture specific proteins or a random pool of the cytosol is debated. On one hand, GFP expressed into the cytosol can be found in extracellular vesicles proportionally to its expression (Mathieu et al., 2021). On the other hand, certain proteins such cyclin D1 appear to be concentrated in extracellular vesicles in a mechanism relying on Hsc70 (Song et al., 2021). In any case, targeting to intraluminal vesicles and in consequence to extracellular vesicles lacks the identification of specific signals. In addition to cytosol, late endosomes are able to capture elements of the mitochondria *via* mitochondria-derived vesicles (McLelland et al., 2016), of endoplasmic reticulum, the Golgi apparatus and autophagosomes (Jeppesen et al., 2019; Wojnacki et al., 2020; Nüchel et al., 2021). Cytosolic misfolded proteins were shown to be captured by endoplasmic reticulum protein USP19, then transferred to late endosomes for secretion (Lee et al., 2016). It is not yet clear if these membranes are engulfed into nascent intraluminal vesicles or if they fuse with the limiting membrane of late endosome and then are incorporated into intraluminal vesicles. Of note, secretion of extracellular vesicles is necessarily accompanied by the release of the soluble content of late endosomes, and this is not yet well characterized. Secretion of extracellular vesicles might be regulated by calcium in certain cells but not all, and several studies suggest that mTOR inhibitors can stimulate this release (Wojnacki et al., 2020; Nüchel et al., 2021).

SNAREs (Soluble *N*-ethylmaleimide-sensitive factor attachment proteins receptors) are the key components of the intracellular membrane fusion machinery. Classically, SNAREs have been divided into two categories: v-SNAREs and t-SNAREs. As the name suggests, v-SNAREs are present on the transport vesicles whereas t-SNAREs are present on the target membrane (Jahn and Scheller, 2006; Südhof and Rothman, 2009).

**FIGURE 2:**
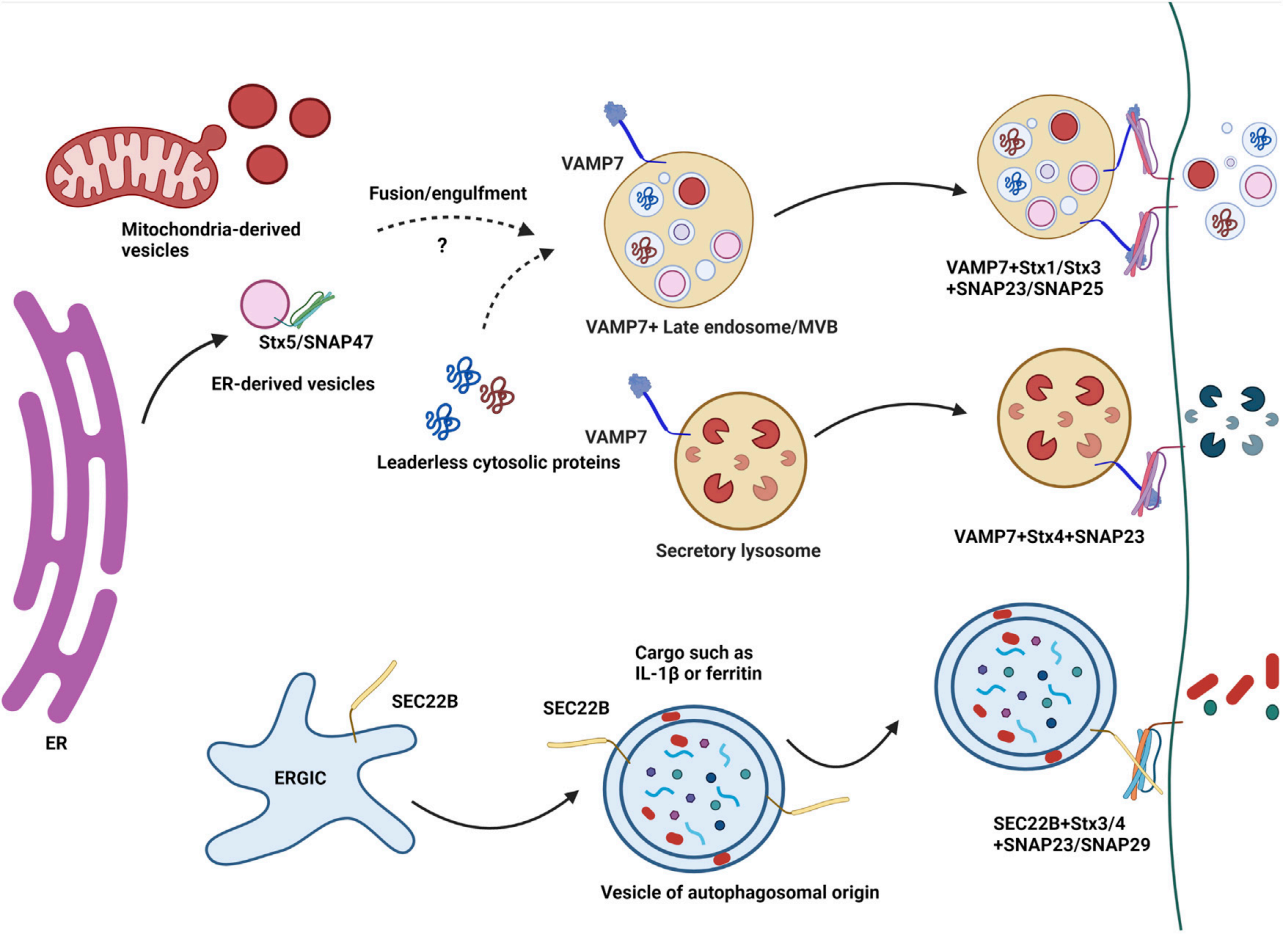
A simplified model of the role of SNAREs in unconventional secretion. Unconventional secretion of extracellular vesicles has been shown to depend on v-SNAREs such as VAMP7 and SEC22B. ER-derived vesicles and mitochondria-derived vesicles may merge with VAMP7^+^ late endosomes/MVBs in a SNARE dependent manner involving VAMP7, Stx5 and SNAP47. Lysosomal exocytosis i.e. the unconventional secretion of lysosomal contents upon the fusion of lysosomes with the plasma membrane involves VAMP7 along with t-SNARES Syntaxin4 and SNAP23. Vojo Deretic’s group has shown the involvement of SEC22B with SNAP23 or SNAP29 and PM SNAREs Syntaxin3/4 in the unconventional secretion of leaderless proteins such as IL-1β and ferritin*.*

Unconventional secretion of extracellular vesicles has been shown to depend on v-SNAREs such as VAMP3, VAMP7 and SEC22B in several studies in different cell types which we discussed below and is depicted in Figure 2. The members of the vesicle-associated membrane protein (VAMP) family have varied intracellular location. VAMP2 has been shown to be present at secretory granules and synaptic vesicles, VAMP3 at secretory granules and early endosomes, VAMP4 in the trans-Golgi, VAMP8 is present on recycling endosomes and also shares its location on late endosomes/MVBs along with VAMP7 (Steegmaier et al., 1999; Chen and Scheller, 2001; Pryor et al., 2004; Marshall et al., 2015; Gordon et al., 2017).

VAMP7, is a SNARE which is insensitive to tetanus and botulinum neurotoxins (hence its other name TI-VAMP for Tetanus neurotoxin Insensitive Vesicle-Associated Membrane Protein) (Galli et al., 1998). VAMP7 is mainly localized to late endosomes and also to the Golgi apparatus and small peripheral vesicles, and particularly colocalizes with CD63 a marker of secretory late endosomes and lysosomes (Advani et al., 1999; Coco et al., 1999; Pols et al., 2013). A particular point of note is that although VAMP7 and VAMP8 share subcellular location on late endosomes and MVBs as mentioned above, according to the human protein atlas and in several studies (Wade et al., 2001; Sato et al., 2011), the expression of VAMP8 is limited more in epithelial and immune cells and is negligible in the brain whereas the expression of VAMP7 has been observed in all cell types (https://www.proteinatlas.org). VAMP7 has the classical SNARE sequence with a SNARE motif and a C-terminal transmembrane domain but also includes an N-terminal extension which is called Longin domain (Filippini et al., 2001). The Longin domain plays an auto-inhibitory role through intramolecular interaction with the SNARE motif (Vivona et al., 2010) thus controlling the fusogenic activity of VAMP7 (Martinez-Arca et al., 2000, 2003). VAMP7 interacts with SNARE partners located at the plasma membrane: Syntaxin 1, Syntaxin 3, SNAP-23 and SNAP-25, autophagosome including Syntaxin 17 and SNAP-29 and ERGIC SNAP-47 (Alberts et al., 2003; Kuster et al., 2015). In particular, VAMP7/Syntaxin 1/SNAP25 and VAMP7/Syntaxin 3/SNAP-23 mediate the fusion of secretory late endosomes with the plasma membrane (Chaineau et al., 2009). Accordingly, Verweij et al. used CD63-pHluorin as an optical reporter to monitor MVB-PM fusion events and observed an increase in release of CD63^+^ exosomes upon phosphorylation of the t-SNARE SNAP23 by histamine H1 receptor mediated signalling (Verweij et al., 2018). Lysosomal exocytosis is an unconventional secretion of lysosomal contents upon the fusion of lysosomes with the plasma membrane. VAMP7 along with t-SNARES Syntaxin4 and SNAP23 mediates lysosomal secretion in fibroblasts (Martinez et al., 2000; Rao et al., 2004; Proux-Gillardeaux et al., 2007) and lysosomal secretion of ATP in astrocytes (Verderio et al., 2012).

Our latest work showed that, in differentiating neuronal cells, VAMP7-dependent late endosomal secretion is also involved in releasing reticulons and atlastins, components of the endoplasmic reticulum, particularly the short form of reticulon 3 (Wojnacki et al., 2020), all molecules which have been linked to axonal growth and regeneration, and neurodegeneration (Yan et al., 2006; Behrendt et al., 2019). This important result was provided by detailed proteomic analysis of the cell lysate and secretome of WT, autophagy-null ATG5 KO and VAMP7 KO PC12 cells. We found that WT cells released proteins which were significantly less abundant in the VAMP7 KO secretome significantly increased in ATG5 KO (Reticulon/RTN1, CALCOCO1, Atlastin/ATL1, SQSTM1/p62, MAP1LC3B/LC3b, RTN4, MAP1LC3A/LC3a, GABARAP, GABARAPL2, RTN3, ATL3) therefore correlating with decreased neurite growth in VAMP7 KO and increased neurite growth and ramification in ATG5 KO PC12 cells. We did not find any KFERQ containing proteins, i.e., markers of the chaperone-mediate autophagy (CMA) pathway (Kirchner et al., 2019; Sahu et al., 2011), which would be significantly enriched in ATG5 KO and decreased in VAMP7 KO secretome. In conclusion, we found that VAMP7 KO and ATG5 KO, which have opposite effects on neurite growth, had clear opposite effects in the secretion of RTN3 which is related to ER-phagy. This secretion appeared particularly enhanced when degradation by autophagy of the endoplasmic reticulum is blocked, such as in ATG5 KO neuronal cells and upon treatment with autophagy blocker bafilomycin A1. This led us to define secretory reticulophagy as a new VAMP7-dependent secretory activity (Vats and Galli, 2021). Interestingly, our findings also align with a recent paper demonstrating that components of the LC3 conjugation machinery regulate and specify cargo loading and secretion *via* extracellular vesicles. Indeed, Leidal et al., found that RNA-binding proteins get packed into extracellular vesicles (EVs) by binding to LC3 via LC3 interacting regions. They term this secretion as LC3-dependent EV loading and secretion (LDELS) (Leidal et al., 2020). Autophagy dependent secretion of modified histone H3 which can be enhanced upon rapamycin treatment or by hypoxia has also been reported (Sulkowski et al., 2021). Also of note, is the fact that VAMP7 was the only identified v-SNARE bearing an LC3-interacting region (Gu et al., 2019). In conclusion, VAMP7 is strongly connected to autophagy-related UPS. To gain further insights on how ATG5 and VAMP7 might regulate neurite growth, we also carried out lipidomic analysis of WT, VAMP7 KO and ATG5 KO PC12 cells. In ATG5 KO cells, enhanced levels of several glucosylceramides (GluCers) and reduced sphingomyelins (SMs) is in good agreement with previous report on the inhibitory effects of glucosylceramide synthase inhibitor on neurite outgrowth in PC12 cells (Mutoh et al., 1998) and accumulation of ceramides in Arabidopsis upon ATG5 inactivation (Havé et al., 2019). VAMP7 KO cells exhibited reduced levels of phosphatidylethanolamines (PEs) in good agreement with the finding that the ethanolamine moiety of PE derived from phosphatidylserine is actively re-acylated only in PC12 cells undergoing NGF-induced neuritogenesis (Ikemoto and Okuyama, 2000). This result is particularly interesting because LC3 and other ATG8 molecules bind PE (Kabeya et al., 2004; Thukral et al., 2015). It will be critical to further characterize how UPS regulates lipid homeostasis.

A persistent question in the field of UPS particularly, the Type III secretion, is how leaderless proteins are packaged into vesicles of autophagosomal/endosomal origins. A recent work by Zhang et al., has described a protein translocation pathway regulated by transmembrane emp24 domain containing protein 10 (TMED10) which can facilitate the transfer of several leaderless UPS cargos into the ERGIC and furthermore into secretory vesicles by the oligomerization of TMED10 (Zhang et al., 2020). TMED10 interacts with the small GTPase Rab21 and this might regulate packaging and release of UPS cargos (Del Olmo et al., 2019). Interestingly enough, VAMP7 interacts with Vps9 and Ankyrin repeat protein (Varp) (Burgo et al., 2009), an exchange factor for Rab21 (Zhang et al., 2006) and effector of Rab32/38 (Wang et al., 2008). Varp interacts with the closed conformation of VAMP7 (Schäfer et al., 2012). Our lab showed that VAMP7 is the starting point of a molecular network that combines proteins belonging to the main classes involved in vesicular trafficking: Varp, kinesin 1 (Kif5A), a molecular motor partner of Varp, GolginA4, a Golgi attachment factor partner of Varp, and the spectraplakin MACF1, an effector of Rab21 (Burgo et al., 2012), which binds both actin and microtubules. We found that this network can send VAMP7 vesicles to the cell periphery along microtubules, thus allowing exocytosis (Burgo et al., 2012; Wang et al., 2018). Varp also interacts with Vps29, a retromer complex subunit involved in Alzheimer disease (Shannon et al., 2014), and this interaction mediates its endosomal membrane targeting. Interestingly, transport of GLUT1 from endosomes to the cell surface requires Varp, VPS29, and VAMP7 and depends on the direct interaction between VPS29 and Varp (Hesketh et al., 2014). Recent work on GRASP55-dependent unconventional secretion also provides strength to the notion that late endosomes and autophagosomes are connected and that secretion might involve a mixed content (Nüchel et al., 2021). GRASP-55 further appeared in the proximome of VAMP7 (Hesketh et al., 2020). Synaptotagmin 7 was found as a VAMP7 partner (Rao et al., 2004) and it is involved in exosome secretion (Hoshino et al., 2013). In conclusion, at least some members of the VAMP7 interactome such as Rab21, GRASP-55 and Synaptotagmin7 appear to be involved in unconventional secretion as discussed above. The detailed molecular mechanisms still require investigation.

Owing to the potential functional redundancy in post-Golgi v-SNAREs, constitutive secretion from the Golgi was shown to be unaffected by depletion of VAMPs 3, 4, 7, 8, and YKT6 individually or in combination in human cells (Gordon et al., 2010). However, in *Drosophila*, depletion of YKT6 caused partial inhibition and the combinatorial depletion of YKT6 and VAMP3, an almost complete block in constitutive conventional secretion (Gordon et al., 2017). Interestingly, YKT6 is also required for the secretion of Wnt proteins in exosomes (Gross et al., 2012) and VAMP3 is involved in the exosome secretion evoked by FGF-2 (Kumar et al., 2020). VAMP3 and SNAP23 were also shown to be involved in the unconventional secretion of tissue transglutaminase in mouse fibroblasts and human endothelial cells (Zemskov et al., 2011). How the functions of YKT6 and VAMP3 in both conventional and unconventional secretions might or not be coordinated will require further investigation. Another SNARE of importance in unconventional secretion is SEC22B. SEC22B, is a longin v-SNARE involved in ERGIC trafficking (Jahn and Scheller, 2006). SEC22B with SNAP23 or SNAP29 and PM SNAREs Syntaxin3/4 is involved in the unconventional secretion of leaderless proteins such as IL-1β and ferritin as shown in human immune cells (Kimura et al., 2017).

V-SNARE VAMP8 can also interact with PM SNAREs such as SNAP23 and Syntaxin4, but there is limited evidence to suggest its role in UPS. Pilliod et al. (2020), show that in neuroblastoma cell lines, the overexpression of VAMP8 can decrease the cellular load of mutated tau proteins by increasing their secretion. However, as the authors mention, the expression of VAMP8 in the brain is extremely low and hence they suggest that another v-SNARE might be involved in the secretion of tau in neurons (Pilliod et al., 2020). Whether this v-SNARE could be VAMP7, however, remains to be proven experimentally. Recently, an alternative protein quality mechanism to tackle misfolded protein was proposed by Lee et al. This pathway termed as misfolding-associated protein secretion (MAPS) is dependent on the ER associated deubiquitylase USP19 and is involved in the unconventional secretion of cytosolic misfolded proteins. They further show the involvement of late endosome resident SNAREs VAMP7 and VAMP8 in this secretion (Lee et al., 2016). The function of VAMP8 in the fusion of autophagosome with lysosome is very clear. This membrane fusion involves Stx17 and SNAP29 as t-SNARE (Diao et al., 2015; Huang et al., 2021). SNAP29’s role in this membrane fusion mechanism is negatively regulated by O-GlcN-acetylation (Guo et al., 2014). Interestingly enough we found an inhibitory effect of the overexpression of SNAP-29 on the exocytic functions of VAMP7 (Kuster et al., 2015). Altogether, this suggests that SNAP-29 expression and regulation might play a central role in the balance between degradative autophagy (which involves VAMP8) and autophagic secretion (which involves VAMP7).

In conclusion, several v-SNAREs (VAMP7, VAMP3, YKT6, SEC22B) might be involved in unconventional secretion of EVs and that might depend on the cell type and signalling mechanisms. Nevertheless, compelling evidence point to VAMP7 and SNAP-23 as central v- and t-SNAREs in this process.

## Role of Unconventional Secretion in Health and Disease

The characterization of the role of unconventionally secreted proteins in shaping the physiological and pathological cellular microenvironment is still developing. Indeed, tumor microenvironment has emerged as a main feature in cancer initiation and progression (Anderson and Simon, 2020). In addition, non-neuronal cells which contribute to the neuronal microenvironment as much as neurons have been implicated in neurodegeneration (Phatnani and Maniatis, 2015). Secreted small molecules and metabolites, nucleic acids, diffusible proteins and extracellular vesicles, are all components of the secretome, which is a source of biomarkers (Uhlén et al., 2019). The secretome can represent the cellular microenvironment in health and disease, as exemplified in the case of the senescence-associated secretome which appears as an indicator of age and medical risk (Schafer et al., 2020).

### Unconventional Secretion in Cancer

A cancerous mass typically consists of heterogenous cancerous cells as well as resident and infiltrating host cells. This entire mixed population of cells can secrete factors either in a conventional or unconventional manner in the extracellular space. Hence, when we talk about cancer secretome, it most likely includes proteins secreted by both cancerous and non-cancerous cells. These tumor cells, host stromal cells, secreted factors and extracellular matrix proteins together make the tumor microenvironment. The fate of cancer progression is largely dependent on interactions between tumor and host cells and the secreted factors secreted by them facilitates this intercellular communication. Hence, cancer secretomes can be of great potential interest as putative therapeutic targets. Apart from mediating interaction with host stromal cells, the secreted factors aid in recruitment of vascular endothelial cells, infiltrating immune cells and cancer associated fibroblasts (Paltridge et al., 2013). Tumor cell secretome is comprised of cytokines, growth factors, enzymes, glycoproteins and extracellular vesicles. Proteomic studies have shown the tumor cell secretome to be markedly different from healthy cell secretome. Thereby, it has been envisioned that cancer secretomes can be a treasure trove of potentially specific cancer biomarkers and can aid in cancer screening and detection (Xue et al., 2008; Mustafa et al., 2017; Madden et al., 2020). Secreted proteins can affect self or adjacent cells or nearby tissues in an autocrine, paracrine or endocrine manner. Tumor cell secretion can induce malignant transformation of normal epithelial cells nearby. Vascular endothelial growth factor (VEGF) secreted by tumor cells plays a role in angiogenesis and enhancing vascularization (Barbera-Guillem et al., 2002). Secreted Epidermal growth factor (EGF) and Transforming growth factor-β (TGF-β) triggers signaling pathways such as PI3K/Akt and Ras/Raf/MAPK which aid in progression of cancer (Larue and Bellacosa, 2005). Matrix metalloproteinases which digest extracellular matrices help with tumor invasiveness and migration while secreted cytokines enhance inflammation by recruiting inflammatory cells (Zucker and Vacirca, 2004; Paltridge et al., 2013). The communication between neurons and tumor cells including glioblastoma cells was proposed to play an important role owing to the release of small molecules like glutamate (Takano et al., 2001; Venkataramani et al., 2019) and serine (Banh et al., 2020).

Exosomes have been shown to be important in cancer and they are also thought to have therapeutic interests, which have already been recently reviewed (Dai et al., 2020). In addition, autophagy is thought to suppress early-stage but to promote late-stage tumor development, a dual effect which might be related to autophagy-dependent paracrine mechanisms thus tumor microenvironment. Because the core of solid tumors is hypoxic therefore under metabolic stress, autophagy is likely to play an important function in tumor initiation and growth (Jin and White, 2008). As pointed in the sections above, VAMP7 acts as a central v-SNARE in regulating unconventional secretion. In specific relation to the above-mentioned molecular and cellular mechanisms of UPS, recent genetic studies particularly using transcriptomics have linked VAMP7 expression to several cancers (Zhu et al., 2020; Wang et al., 2021; Xu et al., 2021; Li et al., 2022). Furthermore, VAMP7 mediates the exosomal release of miR-375 (Kumar et al., 2021), which was involved in glioblastoma and matrix metalloproteases (Steffen et al., 2008). GRASP55 was shown to mediate the release of matrix metalloproteases by late endosomes and autophagosomes (Nüchel et al., 2021) and matrix metalloproteases play a key role in cancer cell invasion and dissemination (Kessenbrock et al., 2010). Interestingly, matrix metalloproteases have been identified in extracellular vesicles (Shimoda and Khokha, 2017). It will be important to characterize the potential role of VAMP7- and GRASP55-dependent unconventional secretion of miRNA and matrix metalloproteases in tumor development. Additionally, regulated lysosomal exocytosis which involves VAMP7 has also been shown to enhance sarcoma progression by exacerbating the release of lysosomal hydrolases (Machado et al., 2015).

### Unconventional Secretion in Neurodegeneration

Extracellular vesicles, particularly small EVs or exosomes have been shown to have an important role in the propagation of neuropathology (Vassileff et al., 2020). Lee et al., reported a misfolding associated protein secretion pathway which uses deubiquitylase USP19 to export misfolded cytosolic proteins (Lee et al., 2016). Defects in protein quality control is a central cause in several neurodegenerative diseases. Parkinson’s disease is characterized by protein degradation, endolysosomal and mitochondrial dysfunctions including autophagy impairments. Familial and sporadic forms of Parkinson’s disease involve mutations in PARK genes like LRRK2, PRKN, VPS35, SNCA which can affect many cell types but seem to lead to the death of only dopaminergic neurons in the brain (Panicker et al., 2021). Non-cell autonomous mechanisms related to the secretome particularly involving astrocytes (Di Domenico et al., 2019) could be part of the complex physiopathology of Parkinson’s disease.

There are several reports of aggregate prone proteins getting secreted in the extracellular space following the Type III UPS. Increasing lysosomal exocytosis can protect human dopaminergic neurons from alpha-synuclein toxicity by releasing it in the extracellular space (Tsunemi et al., 2019). Mutant huntingtin (mHtt), a protein whose aggregation results in Huntington’s disease has also been shown to be secreted via late endosomal/lysosomal unconventional secretion (Trajkovic et al., 2017). The phosphorylation of mHtt at S421 also affects the intracellular transport of VAMP7 positive vesicles (Colin et al., 2008). Earlier work from our lab shows the transport of amyloid precursor protein and the endogenous GPI anchored cellular prion protein in a VAMP7 dependent manner (Molino et al., 2015; Wang et al., 2018). A recent report also suggests that mHtt is unconventionally secreted in a GRASP55 dependent manner (Ahat et al., 2021). VAMP7-dependent secretion mediates the release of α-syn aggregates (Xie et al., 2021). Late endosomes are important for the clearance of protein aggregates associated with neurodegenerative disease (Filimonenko et al., 2007) but it is not known if this could be dependent on late endosomal secretion. It will be now important to characterize the potential role of VAMP7- and GRASP55-dependent unconventional secretion of protein aggregates and other elements of late endosomes and autophagosomes. Whether the secretory mechanisms are part of the initiation of neurodegeneration, an early event related to the microenvironment of fragile neurons and/or participating in the propagation of prion-like proteins (Rastogi et al., 2021) remain to be explored in details.

## Conclusion: A Perspectival Integrated Vision of Secretion

Compared to synaptic vesicle exocytosis, unconventional secretion of late endosomes is rather slow, likely calcium-independent in most cases, neither quantal nor truly scalable (due to the heterogeneity of late endosomes and ILVs), not sustainable (biogenesis is very complex, no true recycling mechanism unlike synaptic vesicles) and possibly even serendipitous regarding the intraluminal vesicles’ capture of cytosol (in the absence of a specific targeting mechanism, there might only be a concentration mechanism for certain cargoes). One might even think that unconventional secretion utilizing late endosomes, for all these shortcomings might represent a primitive form of secretion. From this point of view, it is now rather critical to have an evolutionary perspective on secretory mechanisms: how, when did the different modes of secretion appear during evolution and which are the protein ancestors mediating the basic mechanisms.

In this review, we attempt to focus on the vesicle mediated Type III UPS in which several v-SNAREs are involved. We take an in-depth look at the current body of work which contribute towards delineating the role of VAMP7 in unconventional secretion. Owing to the important role played by unconventional secretion in health and disease, we believe that further explorations into the mechanistic details of the VAMP7 mediated unconventional secretion can provide a clearer view of its impact on cellular physiology and pathology.
